# Melatonin: an anti-tumor agent for osteosarcoma

**DOI:** 10.1186/s12935-019-1044-2

**Published:** 2019-11-29

**Authors:** Hadis Fathizadeh, Hamed Mirzaei, Zatollah Asemi

**Affiliations:** 1 Department of Microbiology, Kashan University of Medical Sciences, Kashan, Islamic Republic of Iran; 2 Research Center for Biochemistry and Nutrition in Metabolic Diseases, Institute for Basic Sciences, Kashan University of Medical Sciences, Kashan, Islamic Republic of Iran

**Keywords:** Osteosarcoma, Melatonin, Therapy

## Abstract

Osteosarcoma is the most common bone tumors which consisted of malignant mesenchymal cells generating osteoid and immature bone. It has been showed that osteosarcoma is common in children and adolescents and shows high mortality rate. A variety of therapeutic approaches (i.e., resection surgery, combined with chemotherapy and radiotherapy) have been used as conventional treatments in patients with osteosarcoma. Despite several attempts to improve therapeutic response, the rate of survival for osteosarcoma has not changed during the past 3 decades. Therefore, the discovery and developing new effective therapeutic platforms are required. Along to the established anti-cancer agents, some physiological regulators such melatonin, have been emerged as new anti-cancer agents. Melatonin is an indolamine hormone which is secreted from the pineal glands during the night and acts as physiological regulator. Given that melatonin shows a wide spectrum anti-tumor impacts. Besides different biologic activities of melatonin (e.g., immunomodulation and antioxidant properties), melatonin has a crucial role in the formation of bones, and its deficiency could be directly related to bone cancers. Several in vitro and in vivo experiments evaluated the effects of melatonin on osteosarcoma and other types of bone cancer. Taken together, the results of these studies indicated that melatonin could be introduced as new therapeutic candidate or as adjuvant in combination with other anti-tumor agents in the treatment of osteosarcoma. Herein, we summarized the anti-tumor effects of melatonin for osteosarcoma cancer as well as its mechanism of action.

## Introduction

Osteosarcoma is known as the most common aggressive and malignant tumor from bone. It usually occurs in the metaphysis of long bones and happens predominantly in children and adolescents [1]. Although the causes of osteosarcoma have not yet been fully elucidated, there is probably a correlation between the rate of bone growth during puberty and the incidence of this disease [2]. It has also been seen that young patients with osteosarcoma are usually taller than healthy subjects in similar age groups [1, 3]. Osteosarcoma accounted for approximately 5% of childhood cancers and also almost 8.9% of childhood cancer-related deaths. The incidence of osteosarcoma is estimated at 5 per million people [4]. Ewing sarcoma is another type of secondary bone cancer which occurred more often in adolescents [5]. In this kind of cancer, the survival rate is about 50% in 5 years after the initial diagnosis [6].

Conventional therapies for osteosarcoma consisted of a combination of surgery and adjuvant and neoadjuvant chemotherapy [7]. Given that before utilization of chemotherapy, less than a 20% survival rate in high-grade conventional osteosarcoma even with surgical amputation, revealing the existence of micrometastases before surgery [8]. The low grade could be treated with excision alone and chemotherapy is avoided if final pathology confirms low grade [8].

Treatments that are used for bone cancers are not associated with effective results and are not able to increase the survival rate [9]. On the other hand, the used therapeutic platforms are related to various side effects [10]. Therefore, novel therapeutic approaches with less or no adverse effects are needed in the treatment of osteosarcoma patients [11].

Melatonin, *N*-acetyl-5-methoxy-tryptamine, plays significant roles in different physiological events such as regulating the sleep–wake cycle, declining of tumor progress, ameliorating of immune system actions, and control of homeostasis in the various tissues [11, 12]. Furthermore, melatonin acts as an antioxidant and oncostatic attributes [13]. Several evidence revealed the repressing effects of melatonin against many types of tumors including breast, endometrium, prostate, ovary, intestine, and liver [14–19]. Bone metabolism is related to the levels of melatonin which lead to the prevention of bone degradation and the progression of bone formation [13]. Given that the reduction of melatonin levels in the specific time points, is associated with high incidence of osteosarcoma. Thus, it seems that melatonin acts as a potential anti-osteosarcoma. Here, we focused on osteosarcoma-related molecular mechanisms affected by melatonin. Moreover, we highlighted the different findings of pre-clinical researches on anti- osteosarcoma effects of melatonin.

### Osteosarcoma pathogenesis: insights in molecular mechanisms

Osteosarcoma is associated with a complex of numerical and structural chromosomal abnormalities and an unstable genome [20]. Many amplifications and deletions occurred in different regions of the genome. For example, 1q23.1–1q21.1, 1p35, 19p13.11–19p13.2, and 6p22.1–6p21.31 amplified whilst 5q14.3–5q22.2, 13q13.2–13p14.3, and 5q12.3–5q13.2 are deleted [21, 22]. It has been showed that these modifications and deletions are related with deregulation of a sequence of molecular targets such as rapamycin (mTOR) and vascular endothelial growth factor (VEGF), cellular adhesion molecules, wingless-type MMTV integration site family (Wnt), and Hedgehog signaling pathways [21]. One of the other aspects of osteosarcoma progression is the inactivation of tumor suppressor genes. For example, the alterations in tumor protein p53 (TP53) and retinoblastoma 1 (Rb1) in sporadic osteosarcoma is approximately 30 to 40 present [20]. The Rb gene mutations resulted in the enhanced function of the transcription factor E2F. In primary osteosarcoma, the high levels of E2F1 have growth- suppressing effects such as p73 induction [23]. The reduced expression of WW domain-containing oxidoreductase (WWOX) in many cancers which acts as a tumor suppressor, is related to aggressive properties and low prognosis [24]. The deletion of the WWOX gene is observed in 30% of osteosarcoma cases which likely is an initial event in osteosarcoma pathogenesis [20].

**Fig. 1:**
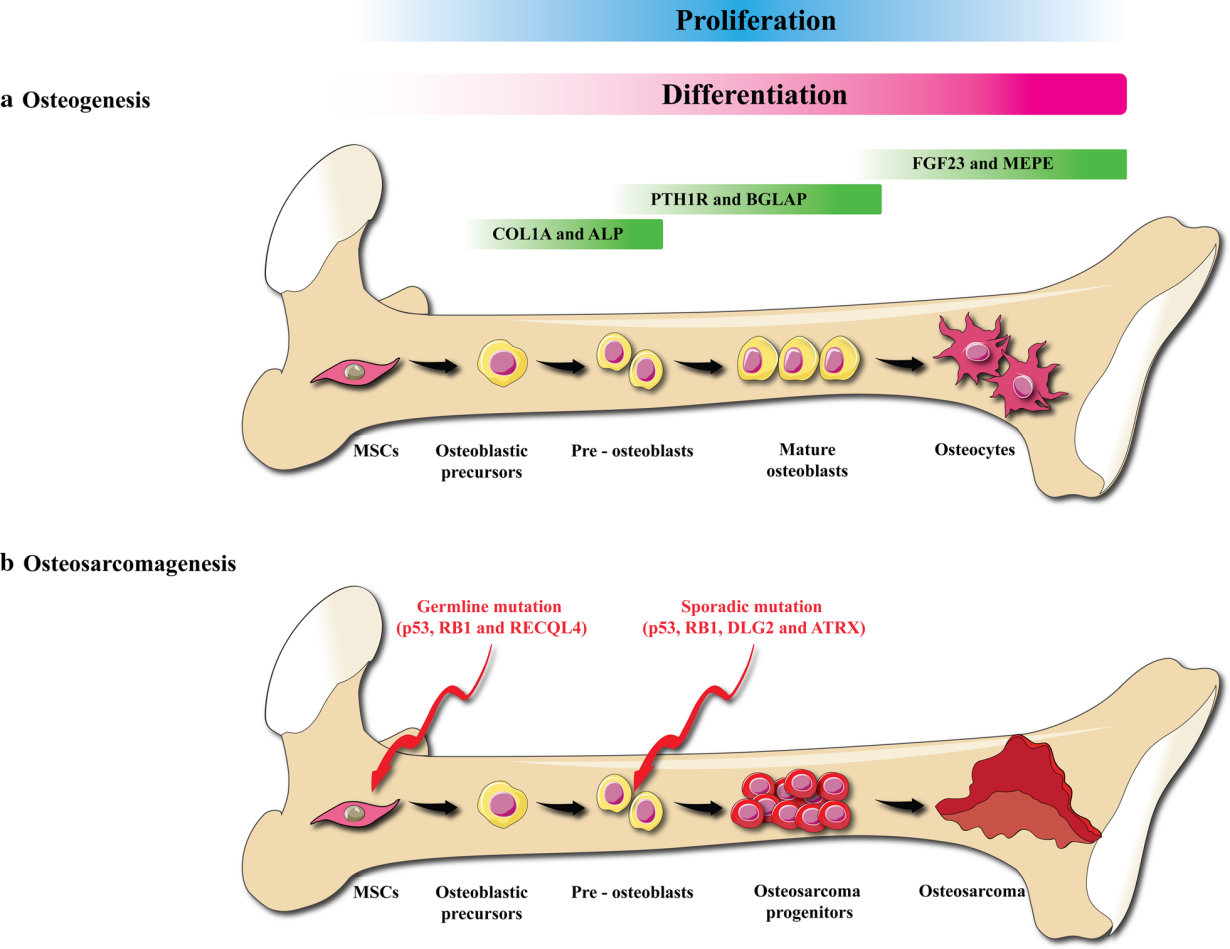
Pathogenesis of osteosarcoma

BCL2-related to athanogene 3 (BAG3) is able to change the interaction between inhibitor of kappa B kinase gamma (IKK-g) and heat shock protein 70 (HSP70) and causes enhancing the accessibility of IKK-g which results in increasing of nuclear factor kappa B (NF-kB) activity and increasing survival [25]. These results suggested that BAG3 could be used as a potential therapeutic candidate in the treatment of osteosarcoma [25]. Bcl-xL downregulation significantly decreased the proliferation of osteosarcoma cells, while its upregulation could increase the cell proliferation [26]. Some studies indicated that the radiosensitivity and chemosensitivity are remarkably enhanced due to the downregulation of Bcl-xL which is related to elevating of caspase-3 function [26]. In osteosarcoma cells, midkine is extremely expressed. The silencing of Midkine induces apoptosis, while recombinant midkine enhances the cell proliferation [22]. One of the signaling networks involved in osteosarcoma is VEGF with a range of 74.1% of expression. So, treatment based on anti-vascular effects may be useful for osteosarcoma [21]. In addition to genetic mutations and damage of cancer suppressor genes, osteosarcoma associated with other factors that promulgate proliferation and metastasis [27]. The TGF-b protein, one member of the superfamily of five isoforms (TGF-b1–5), has a mitogenic efficacy on osteosarcoma cell lines [28]. One of the factors which were identified in the development of osteosarcoma is the change in the insulin-like growth factor-I (IGF-RI) receptor pathway [29, 30]. The IGF-RI, IGF-I/II complex results in a reduction of expression MAPK/ERK and PI3K/Akt/mTOR cascades which promotes migration, proliferation, and survival [31]. A study has reported that overexpression of IGF-I, IGF-IR, and IGF-II in a considerable ratio of osteosarcoma early tumors was observed [32]. Metastatic osteosarcoma is a set of genetic alterations that results in the migration of tumor cells into the bloodstream, inhibition of apoptosis, and proliferate in other tissues [33]. In the Metastasis of osteosarcoma src and Wnt/B-catenin pathways have been involved. Moreover, it was identified a high expression of Notch1 and Notch2 receptors in extremely metastatic osteosarcoma specimens [34]. Also, the elimination of the Fas/Fas ligand pathway leads to apoptosis suppression and developing of osteosarcoma metastases [33]. Figure 1 illustrates different steps from ontogenesis to osteosarcoma.

### Anti-tumor effects of melatonin

**Fig. 2:**
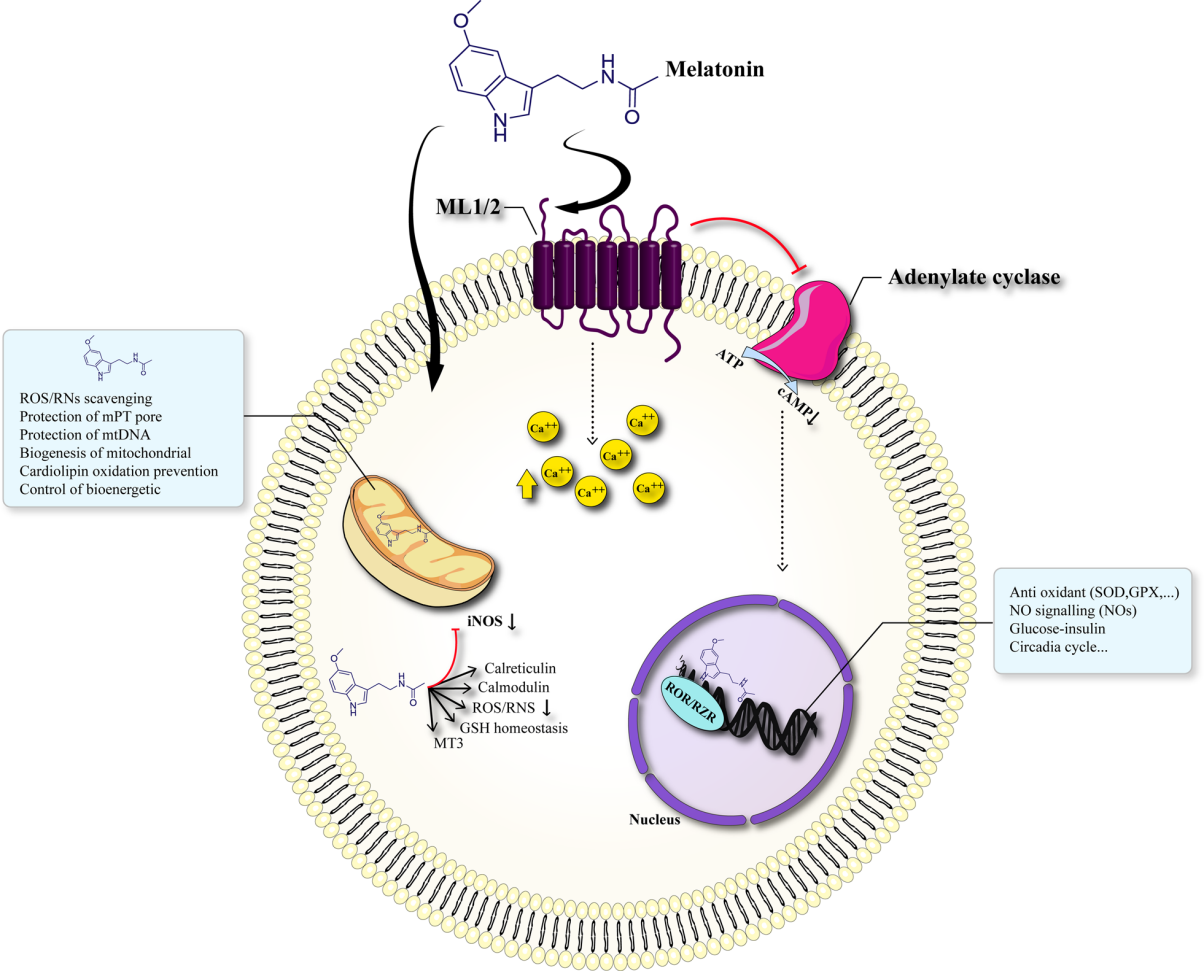
Melatonin and its anti-cancer effects

Melatonin is an indoleamine which secreted by the pineal gland and likely other organs [35]. Melatonin can have multiple functions through receptor-dependent mechanisms and vice versa [36]. It is proven that melatonin has anti-inflammatory and antioxidant activities. Melatonin acts as a scavenger factor and also enables to the regulation of antioxidant enzyme and antioxidant enzyme activity [37]. It has been made clear that melatonin possesses a set of anti-cancer properties including antioxidant, cytostatic, anti-proliferative, pro-apoptotic, and different functions related to its capacity to control epigenetic responses (Fig. 2) [38–41]. In vitro investigations reported that melatonin prevents proliferative ERK1/2 signaling which is a pathway in the modulation of cell division [42]. Notably, melatonin results in activation of ERK1/2 signaling in natural cells, on the contrary, it is able to inhibit ERK1/2 in tumor cells, preventing proliferation and decrease the resistance to cancer chemotherapy [43].

Another pre-clinical study, supported the melatonin exerts a cytostatic effect which lead to the cell accumulation in the G0/G1 phase or delaying in the entry to the S phase [44]. Shen et al. [45] demonstrated that melatonin induced the number of cells in the G1 phase but diminished those in the S phase. Another study was shown the cycle detention and apoptosis occurred after the administration of melatonin in the HepG2 cell line [46]. An important aspect of the anti-tumor effect of melatonin is its ability to induce apoptosis. Melatonin induces apoptosis just in tumor cells and leads to decreasing tumor cell size [47]. In a study on ovarian cancer, it was shown that melatonin causes increasing expression levels of the cleaved caspase 3, Bax, p53, and decreasing expression levels of Bcl-2, thus progresses apoptosis [48]. Wang J et al. [15] evaluated the effect of melatonin on MDA-MB-361 breast cancer cells. Results indicated that melatonin had pro-apoptotic effects which were accompanied via increasing of APAF 1 expression by simultaneously repressing the p300/NF-κB, COX-2/PGE2, and PI3K/Akt signaling. Melatonin is able to induce the releasing of cytochrome c and stimulated activities and cleavage of caspase 9 and 3.

In another study, it was observed that caspase 3, 9, 6, and 7 were activated and melatonin also increased the Bax expression levels and induces the liberation of cytochrome c [49]. Recently, efforts have shifted to discover the role of melatonin in the prevention of tumor metastasis [50]. In several types of cancer, it has been indicated that melatonin has also significant anti-metastatic effects such as its capacity to prevent the epithelial-to-mesenchymal transition (EMT) [51]. The modulation of cell–matrix, cytoskeletal reorganization, and prohibition of angiogenesis are other anti-metastatic mechanisms affected by melatonin [52–54]. The epidermal growth factor receptor 2 (HER2) alongside Mapk/Erk signaling leads to the enhancement of metastasis and invasiveness of cancer cells. Utilization of melatonin considerably decreased the function of Mapk/Erk signaling [41]. Also, melatonin exerts its anti-invasive activity though down regulation of the p38 pathway and detention of activity and expression of metalloproteinases-2 and -9 [55]. Other beneficial actions of melatonin in cancer therapy are including reduction of oxidative stress and toxicity due to chemotherapy and radiotherapy [56]. In addition, emerging data is showing that melatonin shows weak or no anti-cancer effects [57–59]. These studies indicated that melatonin could not be used as first line therapeutic agent in the treatment of various cancers. These studies recommended that melatonin could be employed as adjuvant therapy in combination with primary treatment agents and platforms.

### Melatonin effects on bone cancer

The effects of melatonin on bone cancers open attractive horizons for many researchers. In this regards, several experimental studies have been conducted to assess the underlined mechanisms affected by melatonin in bone cancers. It is proven that bone metabolism is associated with melatonin levels. Melatonin has an effect on bone-linked cell proliferation [60, 61]. It has been showed that, in the specific time points, the levels of melatonin are low and it is related to high incidence of osteosarcoma. These finding hypotheses that melatonin may have an inhibitory effects on osteosarcoma cells [62, 63]. Several studies have shown the anti-proliferative effects of melatonin on the MG-63 osteosarcoma cells and have reported that the inhibitory activity of melatonin related to mitogen-activated kinases and AKT signaling [42, 63]. Melatonin interferes with the proliferation of osteosarcoma cells via modulation of various signaling pathways. Melatonin specifically inhibits the phosphoactivation of ERK1/2 [42]. Also, these studies were demonstrated that the inhibitory function of melatonin correlated with the downregulation of cyclin CDK4 and D1 and of cyclin CDK1 and B1 [42, 63].

Liu et al. [42] demonstrated that the combination of melatonin and PD98059, a selective inhibitor, had a duplicated effect for blocking ERK1/2 activity. One of the mechanisms affected by melatonin to suppress osteosarcoma cells is the induction of apoptosis. Garcia-Santos et al. [64] observed that melatonin could induce apoptosis in the Ewing’s sarcoma cell line. Similarly, an in vitro study reported that melatonin led to the induction apoptotic cell death by applying the Fas/FasL system in Ewing’s sarcoma. Various studies reported that melatonin upregulated the death receptor Fas and its ligand FasL [64]. This study also revealed that melatonin could transiently increase the number of intracellular oxidants and also activate the redox-regulated transcription factor NF-kB [64]. Another study revealed that the combination of melatonin and vincristine could provide an acceptable synergistic effect and induced apoptosis via affecting on the extrinsic pathway in SK-N-MC cells. As well as, there was a significant enhancement in expression levels of caspase-3, -8, -9 and Bid when melatonin/vincristinein complex is used compared to the single treatments [6].

Another mechanism impacted by melatonin which can induce cell death, is SIRT1 inhibition [65]. SIRT1 is a nicotinamide adenine dinucleotide (NAD)-dependent deacetylase which plays an important role in carcinogenesis via deacetylation of main regulatory proteins such as p53 [66]. Cheng et al. [11] suggested that melatonin can inhibit cell growth in osteosarcoma through down-regulation of SIRT1 signaling. Thus, the down-regulation of SIRT1 can be a therapeutic option for treatment of osteosarcoma. Metastasis is very common cancer-related process that there are approximately half of osteosarcoma patients [67]. A recent experimental study reported that melatonin strongly suppresses the invasion and migration of osteosarcoma cells. In addition, melatonin remarkably prohibits the sarcosphere organization of stem cells of osteosarcoma and regulates EMT markers of osteosarcoma cells [68]. SOX9 as a very important transcription factor has a significant role in bone growth [69]. In vivo model showed that melatonin can significantly prevent the initiation and metastasis of osteosarcoma by downregulation of the SOX9-mediated signaling pathway [68]. In a study showed that melatonin could suppress C–C motif chemokine ligand 24 (CCL24) genes’ expression in U2OS and HOS cell lines. The manipulation of CCL24 levels affects osteosarcoma cells motility, invasion, and migration. Authors documented that melatonin was decreased the level of chemokine CCL24 via inhibition of the JNK pathway and subsequently preventing osteosarcoma invasion. In this regards, it seems that melatonin has potential therapeutic candidate in the treatment of metastatic osteosarcoma [70]. Taken together, these results proposed that melatonin exerts anti-tumor effects against osteosarcoma using various pathways and mechanisms (Table 1).

**Table 1:** Experimental studies that investigated the role of melatonin in bone cancer

Type of bone cancer	Form of melatonin	Dosage	Model	Findings	Ref
Osteosarcoma	Melatonin loaded nanoparticles (PLGA)	3.9–500 mg/mL	In vitro (MG-63 cells)	Inhibition of MG-63 proliferation and decrease cell viability	[71]
Ewing sarcoma	Melatonin combined with vincristine	50 µm–1 mM	In vitro (SK-N-MC cells)	Significant enhancement in the activation of Bid and caspase-3, -8, -9	[6]
Osteosarcoma	Melatonin	1000 mM	In vitro (cell line SOSP-9607)	Reductions in GSH, tumor cell vitality, migration ability, adhesion ability, up-regulated acetylated-p53 and down-regulated SIRT1 and increase in the apoptotic index	[11]
Osteosarcoma	Melatonin	4 mM–10 mM	In vitro (MG-63 cells)	Inhibition of the MG-63 proliferation and downregulation of cyclin D1,B1,E and CDK4, CDK1, CDK2	[63]
Osteosarcoma	Melatonin/PD98059 and melatonin	4 mM	In vitro (MG-63 cells)	Prohibition of the proliferation by significantly inhibited phosphorylation of ERK1/2	[42]
Osteosarcoma	Melatonin	0.25–2.0 mM	In vitro (U2OS cells)	Inhibition of the JNK pathway and reduction chemokine CCL24	[70]
Ewing sarcoma	Melatonin	1 mM	In vitro (A-673, TC-71, A-4573 cells)	Reversion ewing sarcoma metabolic profile that was related to its cytotoxicity	[72]
Ewing sarcoma	Melatonin	1 mM	In vitro (SK-N-MC, TC-71 A673, SK-ES1, A4573)	Expression of Fas and its ligand Fas L, activation of the redox-regulated transcription factor Nuclear factor-kappaB and enhance in intracellular oxidants	[64]

## Conclusions

Osteosarcoma is known as high-grade primary bone malignancy. Despite the many available anti-tumor therapies, the 5-year survival rate is only 60–70%. The different strategies are used to treat osteosarcoma including the targeting of the altered transcription factors, cell proliferation regulators, apoptosis targets, and angiogenesis modulators, to date, due to the side effects related to the established treatments, researchers try to find new or develop therapeutic platforms to overcome to these current limitations. Due to the parallel incidence of osteosarcoma and melatonin levels, this hormone could be introduced as a new candidate for the treatment of osteosarcoma. Several experimental studies have reported the anti-cancer effects of melatonin against osteosarcoma. The results showed that melatonin can exert its anti-tumor properties through activation/inhibition various mechanisms such as induction of apoptosis, anti-proliferative, and anti-oxidant activities. Also, observing the positive effects of melatonin on preventing of the invasion and migration of osteosarcoma cells to other organs is a promising therapeutic strategy to the prevent osteosarcoma metastasis. Collectively, melatonin alone or in combination with other therapeutic agents may be a good option for osteosarcoma cancer treatment.

## Data Availability

The primary data for this study is available from the authors on direct request.

## Abbreviations

VEGF: vascular endothelial growth facto
NF-kB: nuclear factor kappa B
